# Iron improves the antiviral activity of NK cells

**DOI:** 10.3389/fimmu.2024.1526197

**Published:** 2025-01-14

**Authors:** Simone Schimmer, Vaasudevan Sridhar, Zelal Satan, Anton Grebe, Mohamed Saad, Bernd Wagner, Nele Kahlert, Tanja Werner, Dana Richter, Ulf Dittmer, Kathrin Sutter, Elisabeth Littwitz-Salomon

**Affiliations:** ^1^ Institute for Virology, University Hospital Essen, University of Duisburg-Essen, Essen, Germany; ^2^ Institute for the Research on HIV and AIDS-associated Diseases, University Hospital Essen, University of Duisburg-Essen, Essen, Germany; ^3^ Department of Clinical Chemistry, University Hospital Essen, Essen, Germany

**Keywords:** natural killer cells, iron metabolism, viral infection, antiviral function, immunometabolism, iron supplementation

## Abstract

Natural killer (NK) cells are innate immune cells that play a crucial role as a first line of defense against viral infections and tumor development. Iron is an essential nutrient for immune cells, but it can also pose biochemical risks such as the production of reactive oxygen species. The importance of iron for the NK cell function has gained increasing recognition. We have previously shown that NK cells require iron to efficiently eliminate virus-infected target cells; however, the impact of nutritional iron deficiency on NK cell function and the therapeutic benefits of iron supplementation remain unclear. Here, we demonstrate that diet-related low iron levels lead to increased retroviral loads due to functional NK cell impairment, while iron supplementation enhances NK cell proliferation, as well as their cytotoxic efficacy. Notably, iron-treated NK cells exhibited significant metabolic changes, including mitochondrial reorganization. Interestingly, although iron supplementation decreased the NK cell’s cytokine production, it significantly improved NK cell degranulation and the expression of cytotoxicity-associated proteins. These findings highlight the critical role of iron in maintaining NK cell immunity and suggest that iron supplementation may hold therapeutic potential for supporting the treatment of viral infections and immunodeficiency disorders.

## Introduction

Natural Killer (NK) cells are a critical component of the innate immune system, known for their ability to recognize and eliminate virus-infected cells and tumor cells without prior sensitization. They play a crucial role in the first line of defense by producing cytokines such as Interferon (IFN)γ and Tumor necrosis factor (TNF) and mediating cytotoxicity through the release of granules filled with Granzymes (Gzms) and Perforin (1). NK cells are equipped with activating and inhibitory receptors that enable them to detect changes in the expression of surface molecules (e.g. major histocompatibility complex I, CD155, antibody-antigen complexes) on target cells, thereby distinguishing between healthy and abnormal cells (2). NK cells become activated by a variety of cytokines such as interleukins and interferons (3, 4). Type I IFNs (e.g., IFNα, IFNβ) are rapidly induced following most viral infections and play a crucial role in antiviral defense. They induce antiviral restriction factors as well as activate innate and adaptive immune responses (5). It has been shown before that especially IFNα11 improves the antiviral function of NK cells during acute FV infection (6). Type III IFNs (IFNλ) are distinct from type I IFNs, but they also share some functional similarities in antiviral defense. IFNλ mainly act on epithelial cells and they play a critical role in defending mucosal surfaces. Their direct effect on NK cells is controversially discussed (7).

In order to fulfil their antiviral functions, NK cells have to reprogram their metabolism to fuel their increased energetic and biosynthetic demands (8–11). This metabolic response involves several key changes, including the upregulation of nutrient transporters, increased expression of metabolic enzymes, and an expansion of mitochondrial mass. Together, these adaptations facilitate enhanced flux through critical metabolic pathways, particularly glycolysis and oxidative phosphorylation (OXPHOS). As a result, there is an increase in energy production in the form of adenosine triphosphate (ATP), along with an enhanced biosynthetic capacity. This metabolic reprogramming enables cells to meet heightened energy demands and support the increased production of cellular components necessary for their activated state. We have shown that murine retrovirus but also murine cytomegalovirus (MCMV) infections resulted in increased nutrient uptake, improved glycolysis and oxidative phosphorylation in NK cells (8). In murine viral infections, cytotoxicity and migration of NK cells depend on the breakdown of fatty acids (β-oxidation) for energy generation, thus resulting in sufficient ATP levels for activated NK cells (11, 12).

Beside increased energy demands, NK cells strongly depend on the uptake of iron and are sensitive to changes in iron levels (8). Rodents require approximately 35-250 mg/kg of dietary iron daily, while humans require 7-27 mg per day, depending on factors such as growth, weight gain, and pregnancies (13–15). Iron is an essential micronutrient required for numerous cellular processes, including DNA synthesis, respiration, and metabolic functions (16). Iron is distributed to tissues through the binding to the iron-transport protein transferrin and extracted from blood plasma mainly for hemoglobin synthesis by erythrocyte precursors in the bone marrow (17). Mitochondrial metabolism depends extremely on iron, as essential steps in the tricarboxylic acid cycle (TCA) cycle and electron transport chain rely on iron-sulfur cluster-containing and heme-containing proteins (18). Iron deficiency can impair the function of murine and human NK cells, leading to reduced cytotoxicity and compromised immune responses (8, 19). Conversely, iron overload can be detrimental, generating reactive oxygen species that can damage cells and tissues (20). In addition, iron is also crucial for the survival and virulence of many pathogens, like human viruses (16). Pathogens often hijack the host’s iron metabolism to support their replication and spread. For instance, people living with human immunodeficiency virus (HIV) frequently exhibit altered iron homeostasis, which can exacerbate the infection and impact the efficacy of the immune response (21). Studies have shown that HIV can manipulate iron regulatory pathways to create a favorable environment for its replication, leading to increased viral loads and progression of the disease (22).

Here, we used the murine Friend retrovirus (FV) to analyze the viral loads in mice fed with an iron deficient diet and elucidate the effect of iron supplementation on NK cell responses. FV is a complex composed of two distinct components: 1) Friend Murine Leukemia Virus (F-MuLV), which is replication-competent but apathogenic and 2) Spleen Focus-Forming Virus (SFFV), a replication-defective but pathogenic virus (23). Adult C57BL/6 mice exhibit resistance to FV-induced disease and develop a robust and early immune response that effectively controls viral replication. However, this response is unable to completely eliminate the virus, resulting in a persistent, lifelong chronic infection in mice. FV as well as HIV integrate into the host genome and establish a life-long chronic infection. FV causes erythroleukemia in susceptible mice (e.g. BALB/c) whereas HIV leads to progressive immune deficiency and AIDS in humans (24). Although the pathogenicity of the two viruses in their hosts is quite different, the infection of C57BL/6 mice results in a strong anti-retroviral immune response, which is comparable to responses against HIV in humans in terms of e.g. Tregs (25, 26), cytotoxic CD8^+^ T cells (27), and NK cells (28, 29). Thus, FV infection serves as a valuable model for studying host immune responses during both acute and chronic infections. However, it is not suitable for investigating the full spectrum of pathogenicity associated with human retroviral diseases. NK cells play a crucial role in the immune response against retroviral infections, including FV, simian immunodeficiency virus (SIV), and HIV (29). Specifically, NK cells are important in restricting viral replication during the acute phase of these infections (30). During FV infection, NK cells reprogram their metabolism and increase their oxidative phosphorylation, glycolysis as well as require β-oxidation to fuel the increased energy demands (8, 11). First insights into the importance of iron for NK cell metabolism were gained in FV infection by impairing iron transport into serum through application of the hormone hepcidin (8). This approach provided crucial evidence linking systemic iron levels to NK cell metabolic fitness and functional capacity, highlighting the critical role of iron in NK cell-mediated immune responses. Thus, the FV mouse infection model serves not only as an excellent experimental system for investigating the role of NK cells but also to study the significance of iron metabolism and benefits of iron supplementation on antiviral responses against retroviruses.

Here, we show that the upregulation of the transferrin receptor on NK cells is a type I IFN-induced mechanism. Diet-induced iron deficiency resulted in increased viral loads and decreased GzmB^+^ NK cells, whereas iron supplementation clearly improved the NK cell cytotoxicity and increased the mitochondrial polarization. The insights into how iron supplementation enhances NK cell function during retrovirus infection are crucial for advancing our understanding of antiviral immunity. This knowledge has significant implications for the development of innovative NK cell-based therapies targeting infectious diseases such as HIV.

## Materials and methods

### Mice and diet

The experiments utilized sex- and age-matched inbred C57BL/6 mice from Harlan Laboratories, Germany, which were kept in a pathogen-free environment. The mice were at least 7 weeks old at the beginning of the experiments. Mice were housed under 12:12 light cycle in a relative humidity of 55 ± 10 and a temperature of 22 ± 2°C. Mice were fed ad libitum with control diet (Ssniff, E15510-04, 196 mg/kg of iron) and iron-deficient chow (Ssniff, E15510-24, <10 mg iron/kg) for four weeks followed by FV infection.

### Virus infections

The FV complex, comprising of B-tropic Friend murine leukemia helper virus and polycythemia-inducing spleen focus-forming virus, was administered. The FV stock was created as a 15% spleen cell homogenate from susceptible BALB/c mice infected for 14 days with 4,000 spleen focus-forming units (SFFU) of FV. Mice received intravenous injections of 0.1 ml phosphate-buffered saline (PBS) containing 40,000 SFFU of FV and mice were analyzed 7 days post infection (dpi). Importantly, the virus stock did not contain lactate dehydrogenase-elevating virus. Immortalized mouse embryonic fibroblasts (CIM) were infected with Bac-derived wild type Murine Cytomegalovirus (MCMV) Smith strain for virus propagation. At 3-4 days post infection the cells were then collected and the virus was purified from the infected cells by ultracentrifugation at 27,000 g for 1 hour as previously described (31). The virus titer was determined by performing standard plaque assay on Mouse newborn fibroblasts (MNC) (32). Female C57BL/6 mice, aged 6-12 weeks, were infected intraperitoneally with 2x10^5^ PFU in 100 μl PBS for 8 weeks. Herpes simplex virus 1 (HSV-1) F strain was propagated in CIM cells. At 4-5 days post-infection, the cells and supernatant were harvested, and subjected to 3-4 freeze-thaw cycles to enhance virus yield. The mixture was then centrifuged for 30 minutes, and the resulting supernatant was collected for final purification via ultracentrifugation at 23.000 g for 2 hours. Virus titer was determined using an end-point infection assay (TCID50) on CIM cells. Female C57BL/6 mice, aged 6-12 weeks, were infected intravenously with 5x10^6^ TCID50 in 100 μl PBS for 8 weeks. All experiments were approved and conducted in accordance with the guidelines of the State Agency for Nature, Environment, and Consumer Protection (LANUV).

### Cell lines

*Mus dunni* fibroblast and mouse lymphoma (YAC-1) cell lines were cultured in RPMI 1640 supplemented with 10% fetal bovine serum and 100 U/ml Penicillin/100 μg/ml Streptomycin. Cells were cultured in a humidified atmosphere with 5% CO_2_ at 37°C.

### Infectious center assay

Single-cell suspensions obtained from femur and tibia of hind legs were prepared and serially diluted in 10-fold increments. These dilutions were then co-cultured with *Mus dunni* cells for 72 hours. After incubation, cells were fixed with ethanol. To visualize the infectious centers, cells were immunostained using a F-MuLV envelope-specific monoclonal antibody (clone 720). The F-MuLV envelope-specific antibody was generated in a hybridoma cell line (H720). This primary antibody was then detected using a peroxidase-conjugated goat anti-mouse secondary antibody (Dianova). The staining was developed by incubating the cells with aminoethylcarbazol (Sigma, 0.1 mg/ml). Following development, the cells were washed with water to reveal distinct foci, each representing an infectious center.

### NK cell expansion and IFN treatment

Splenocytes were isolated from naive C57BL/6 mice and cultured for 6 days in 12.5 ng/ml IL-15 (BioLegend). On day 6, IL-2 (20 ng/ml), IL-12 (10 ng/ml), murine IFNα11 (500 U/ml) (18), murine IFNβ (500 U/ml) or murine IFNλ2 (100 ng/ml, Peprotech) were added for 18 hours, if indicated. Cells were incubated in a humidified atmosphere with 5% CO_2_ at 37°C.

### Transferrin uptake assay

Splenocytes were treated overnight with IFNα11 (500 U/ml), IFNβ (500 U/ml), IFNlambda2 (100 ng/ml) and IL-2 (20 ng/ml)/IL-12 (10 ng/ml) or left untreated. Cells were washed twice with serum-free RPMI supplemented with 0.5% BSA. Spleen cells were then incubated with 5 μg/ml of Alexa Fluor 647-conjugated Transferrin (T23366, Invitrogen) for 10 min at 37°C. Uptake was stopped by washing twice with ice-cold acid wash buffer (PBS supplemented with 150 mM NaCl and 20 mM citric acid, pH 5) followed by ice-cold RPMI + 0.5% BSA. Cells were stained for surface markers and analysed by flow cytometry.

### Flow cytometry

Bone marrow cells were flushed from murine tibia and femur into a single-cell suspension and washed. Cells were incubated with fluorochrome-conjugated antibodies for 15 minutes at room temperature, protected from light. After staining, the cells were washed and either analyzed immediately using a multi-parameter flow cytometer or fixed for subsequent intracellular staining using a Fixation/Permeabilization Solution Kit from BD Biosciences.

For cytokine detection, splenocytes were restimulated prior to staining. The cells were incubated for 3 hours at 37°C in RPMI buffer containing ionomycin at a concentration of 500 ng/ml, phorbol myristate acetate (PMA) at 25 ng/ml, monensin at 1X (BioLegend), and brefeldin A at 2 μg/ml. For staining of mitochondria, MitoSpy Green (BioLegend, 1 mM), Tetramethyl-rhodamine, Methyl Ester, Perchlorate (TMRM, Invitrogen; 100 nM) and CellROX (Invitrogen, 5 µM) were added in RPMI supplemented with 1% Pen/Strep (Gibco) and 10% FBS (Sigma) and incubated in a humidified atmosphere with 5% CO_2_ for 20 min at 37°C.

Flow cytometry analysis was performed using several antibodies, including CD3 (clone 145-2C11 from Thermo Fisher), CD69 (clone H1.2F3 from BioLegend), CD71 (clone R17217 (RI7 217.1.4) from Thermo Fisher), CD107a (clone 1D4B from Becton Dickinson), CD11b (M1/70 from Becton Dickinson), CD11c (N418 from Becton Dickinson), F4/80 (BM8 from BioLegend), Gr1 (RB6-8C5 from Thermo Fisher), Granzyme A (GzmA) (GzA-3G8.5 from Thermo Fisher), Granzyme B (GzmB) (clone GB11 from Becton Dickinson), IFN-γ (clone XMG1.2 from Thermo Fisher), KI-67 (clone B56, Becton Dickinson), NK1.1 (clone PK136 from Becton Dickinson), Ter119 (clone TER-119 from BioLegend), TNFα (clone MP6-XT22 from BioLegend), TRAIL (clone N2B6 from BioLegend). To exclude dead cells, cells were stained with Zombie Aqua (BioLegend) or fixable viability dye (Invitrogen).

### Analysis of iron

Murine serum was obtained by spinning the clotted blood sample at 8000×g for 5 min and stored at −80°C. Serum iron was quantified using the Atellica CH 930 Analyzer (Siemens Healthineers) and the Iron_2 and Iron_3 methods at University Hospital Essen, Germany. The Iron_2 and Iron_3 assays are methods for the quantitative determination of iron in serum and plasma used on the Atellica CH 930 systems of Siemens Healthineers in routine operation of the Department of Clinical Chemistry at Essen University Hospital. In both methods, Fe3+ is reduced to Fe2+ by transferrin under acidic conditions and simultaneously with ascorbic acid. In the Iron_2 method, a complex formation by ferrozin with Fe2+ occurs. Quantification is carried out by spectrometric measurement of absorption bichromatically at 571 and 658 nm. In the Iron_3 method, on the other hand, a complex of Ferene and Fe2+ is formed, the absorption of which is measured spectrometrically at 596 and 694 nm bichromatically. In the course of the study, the central laboratory had to switch from the Iron_2 to the Iron_3 assay because the Iron_2 method was no longer available. Internal verifications at the Department of Clinical Chemistry showed an excellent agreement between the two methods with almost the same sensitivity. Both methods can be traced back to the reference material SRM 937 of the National Institute of Standards and Technology (NIST).

### DFO treatment and iron supplementation *in vitro*

Single cells were cultured for three days with IL-15 (12.5 ng/ml, BioLegend) and were treated with DFO (Sigma, 100 µM) for 72 hours. FeSO_4_ (Sigma, 200 µM) was added for the final 18 hours.

If cells were treated with FeSO_4_ only, single cells were plated and freshly prepared FeSO_4_ (Sigma, 200 µM) was added for a total of 18 hours. Cells were incubated in a humidified atmosphere with 5% CO_2_ at 37°C, washed twice and measured at BD Canto II.

### NK cells transfer

Bone marrow cells of FV-infected animals (7 dpi) were treated with FeSO_4_ (Sigma, final concentration 200 µM) or left untreated for 18 hours (37°C) followed by NK cells isolation using magnetic beads. 0.5 x 10^6^ isolated NK cells were transferred into C57BL/6, which were subsequently infected with 40,000 SFFU of FV. Viral loads were determined 3 days post infection by an infectious center assay.

### NK cell proliferation

Bone marrow cells were isolated and stained with Tag-it Violet (BioLegend, 5 µM). Cells were plated into 96 well U-bottom plates. IL-15 (50 ng/ml, BioLegend) and IL-18 (50 ng/ml, BioLegend) were added to cells as well as FeSO_4_ (50 µM, Sigma). Cells were incubated in a humidified atmosphere with 5% CO_2_ at 37°C. After 3 days, proliferation of NK cells was assessed by flow cytometry (BD Canto II) as measured by loss of the Tag It Violet dye.

### Detection of puromycin and mitochondrial dependence using SCENITH

RPMI 1640 medium, supplemented with 10% Fetal Bovine Serum and 100 U/ml Penicillin/100 µg/ml Streptomycin, was prewarmed. Single cells from bone marrow were treated with FeSO_4_ for 18 hours or left untreated, washed, and then resuspended to 96 well plates. Inhibitors were added at final concentrations of 100 mM 2-Deoxy-D-glucose and 1 µM oligomycin, followed by a 30 minutes incubation at 37°C. Subsequently, puromycin was added at a final concentration of 10 µg/ml (Sigma) for 20 minutes. Cells were washed with cold PBS, and FC blockage (BD Biosciences) was performed at 4°C for 5 minutes. Cell surface staining was conducted at 4°C for 30 minutes in the dark, after which the cells were washed. Cells were then fixed using the Fixation/Permeabilization Solution Kit (BD Biosciences). Anti-puromycin staining (Sigma, clone 12D10) was performed in perm-buffer at 4°C for 1 hour. Finally, the cells were analyzed using BD Canto II. Mitochondrial dependency was calculated using the formula: 100 x (no inhibitor – oligomycin)/(no inhibitor – 2-DG Oligo).

### *In vitro* kill assay

Bone marrow cells were treated with or without FeSO_4_ for 4 or 18 hours at 37°C. YAC-1 cells were stained for carboxyfluorescein succinimidyl ester (CFSE, BioLegend, 2.5 µM) and seeded in a 200:1 ratio with single cells from bone marrow. After 4 hours, fixable viability dye was used to determine dead cells. YAC-1 killing was calculated by (dead YAC-1 tumor cells)/(all YAC-1 tumor cells) x 100.

### Statistical analyses

Data visualization and statistical analyses were performed using GraphPad Prism version 8 or BioRender.com. For comparisons between two groups, the Mann-Whitney test was applied for nonparametric data, while the unpaired t-test was used for parametric data. When analyzing multiple groups ordinary one-way ANOVA with Tukey’s multiple-comparison test was employed for parametric data. Additionally, correlations between variables were assessed, and linear regression analysis was conducted to determine the relationship between variables and evaluate the goodness of fit. Spider plots were generated with Excel.

## Results

### NK cell activation correlates with transferrin receptor expression

**Figure 1:**
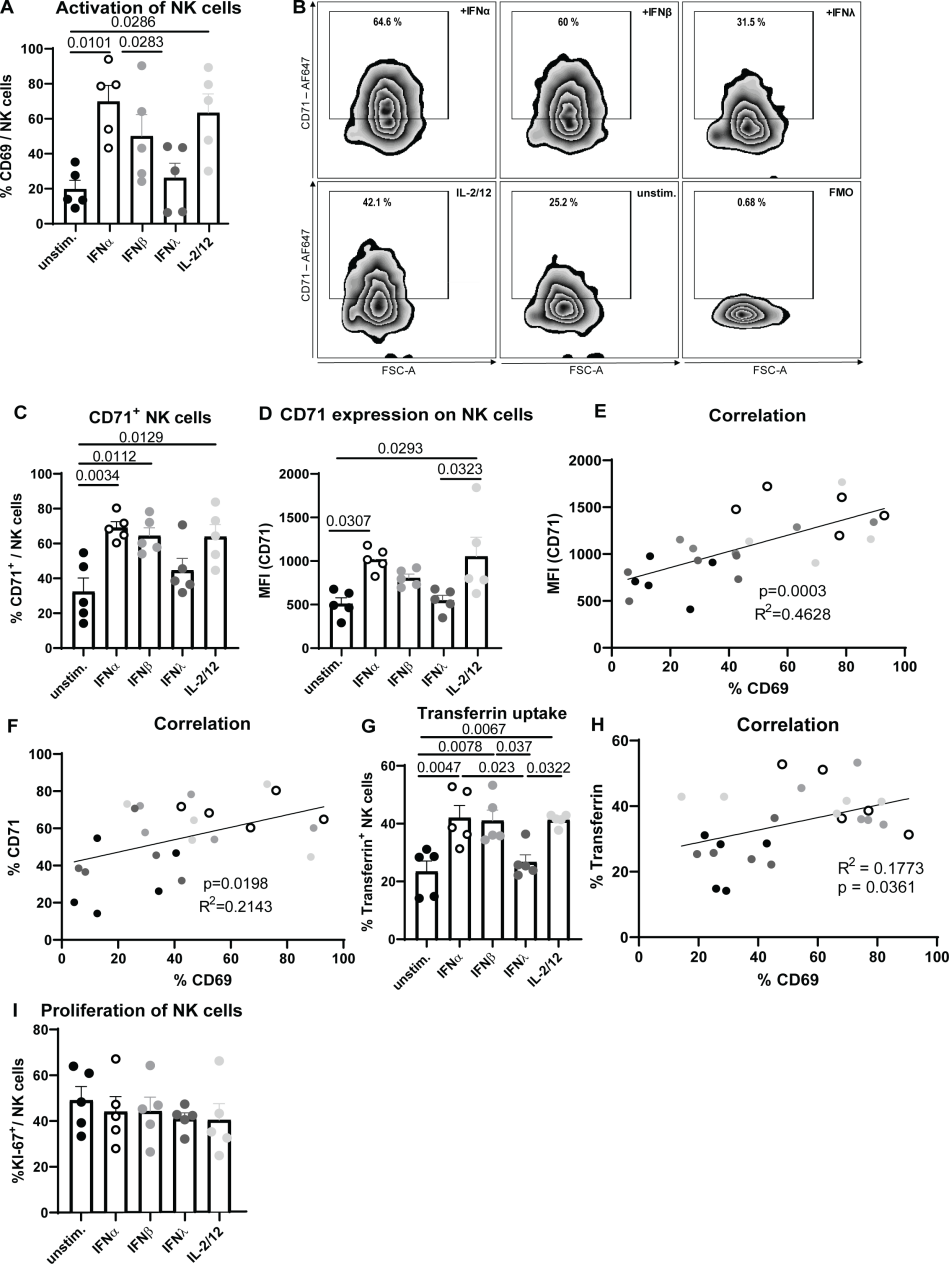
Increased activation and transferrin receptor expression after IFNα stimulation of NK cells. Expanded NK cells were stimulated with IFNα11 (500 U/ml), IFNβ (500 U/ml), IFNλ2 (100 ng/ml), IL-2/12 (20 ng/ml, 10 ng/ml) for 18 hours or left unstimulated (unstim.). NK cells (viable, CD3^-^, NK1.1^+^) were analyzed for activation (CD69) **(A)**. Representative dot plots and bar graphs of CD71^+^ NK cells and transferrin receptor (CD71) expression on CD71^+^ NK cells are shown in **(B–D)**. Statistically significant differences between the groups were analyzed with an Ordinary one-way ANOVA. All data are presented as mean values ± SEM. In **(E, F)**, correlation of CD71 (MFI, %) and CD69 (%) is shown. Splenocytes were stimulated or left untreated overnight and the uptake of transferrin was measured in NK cells by flow cytometry **(G)**. Correlation of transferrin uptake and activation is shown in **(H)**. Five mice from 3 independent experiments were used and analyzed by an ordinary one-way ANOVA. Black circles represent unstimulated, open circles IFNα-, grey IFNβ-, dark grey IFNλ- and bright grey IL-2/-12-stimulated NK cells. In **(I)**, the NK cell proliferation was analyzed by using KI-67. For **(A, C–F)** five mice per group collected from five independent experiments were used (n=5).

Until now, the involvement of IFNs in iron homeostasis, which is vital for the proliferation and function of immune cells, is not known. To understand the role of IFN stimulation on NK cell activation (CD69) and transferrin receptor (CD71) expression, expanded NK cells were treated with murine IFNα11, IFNβ or IFNλ2 and analyzed via flow cytometry (**Figures 1A–F**) (33). IL-2/12 stimulation was used as positive control (34). Stimulation of NK cells with IFNα significantly increased the frequency of NK cell activation (**Figure 1A**) as well as the percentage of CD71^+^ NK cells and CD71 expression (**Figures 1C, D**) similar to IL-2/12-treated NK cells. IFNβ slightly increased the activation and transferrin receptor expression in NK cells, although not significant, whereas IFNλ stimulation had no effect on NK cell activation and CD71 expression. Linking the CD71 expression with the activation of NK cells revealed a positive correlation of both parameters (**Figures 1E, F**). NK cells showed an increased uptake of iron, measured by transferrin uptake, after IFNα11, IFNβ, and IL-2/12 stimulation compared to unstimulated and IFNλ-treated cells (**Figure 1G**). Transferrin uptake of NK cells positively correlated with NK cell activation (**Figure 1H**). To investigate whether the elevated CD71 expression on NK cells following IFNα and IFNβ stimulation was a consequence of increased proliferation and subsequent higher iron demand, we assessed NK cell proliferation using the marker KI-67 (**Figure 1I**). Our analysis revealed no significant differences in the proportion of proliferating NK cells between the experimental groups. These findings suggest that the observed increase in CD71 expression is more likely a direct effect on iron homeostasis rather than a result of enhanced proliferation and demonstrate that type I IFNs, which are rapidly induced upon viral infections, influence the potential of NK cells to take up iron, thus contributing to proper iron supply in NK cell.

### Increased viral replication in diet-induced iron deficiency due to NK cell dysfunction

**Figure 2:**
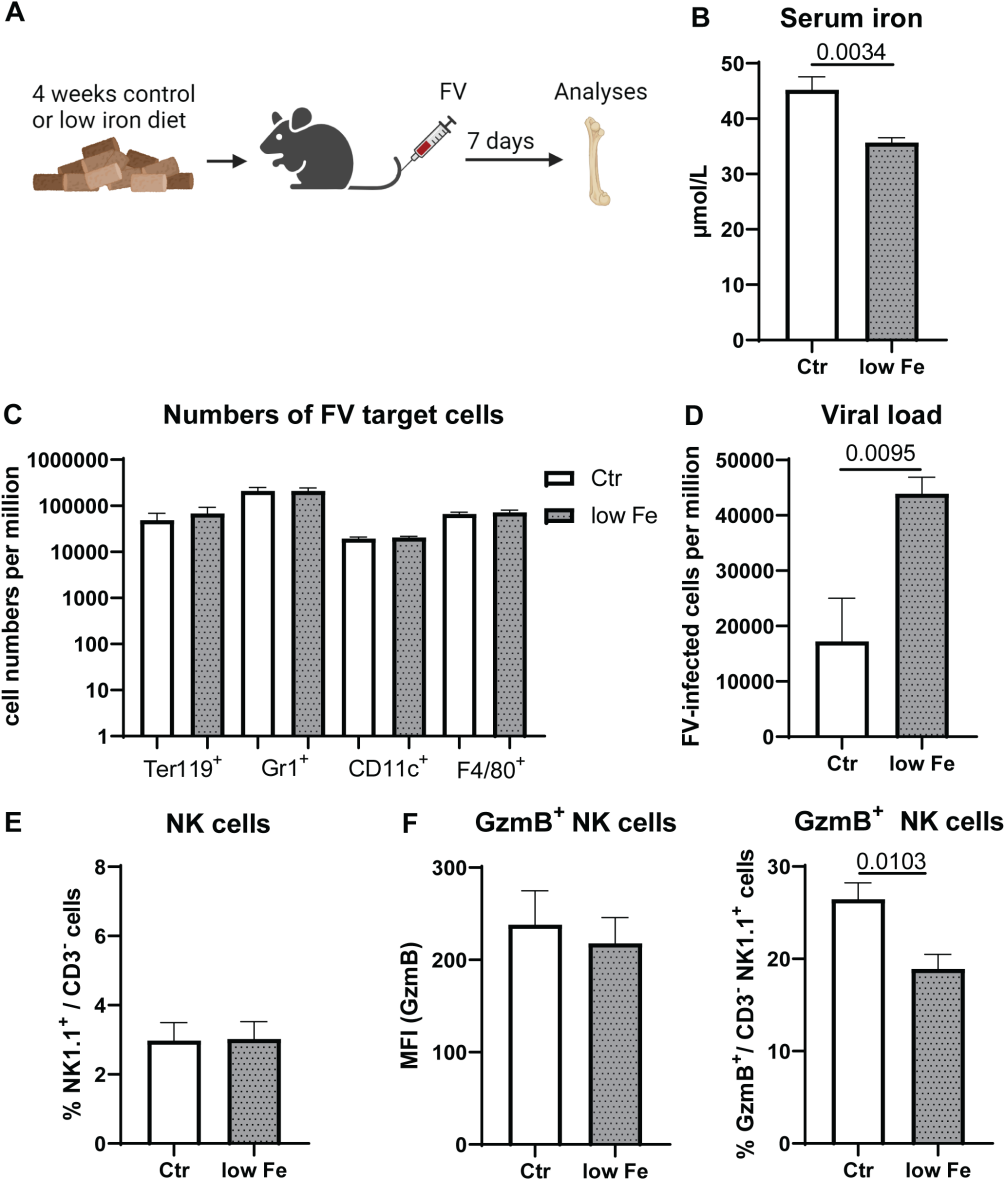
Increased viral replication and reduced frequencies of GzmB^+^ NK cells in mice with low iron diet. C57BL/6 mice were fed with control diet (Ctr, 196 mg/kg of iron) or a low iron diet (low Fe <10 mg iron/kg) for four weeks. Mice were infected with 40,000 SFFU of FV and bone marrow cells were isolated after 7 dpi **(A)**. Serum iron levels were analyzed in both groups **(B)**. FV target cells such as erythroblasts (Ter119^+^), granulocytes (Gr1^+^), dendritic cells (CD11c^+^) and macrophages (F4/80^+^ CD11b^+^) are displayed as numbers per million in **(C)** Viral loads in the bone marrow of control diet and low iron fed mice are shown in **(D)** Frequencies of NK cells were analyzed in **(E)** GzmB expression of NK cells and percentage of GzmB^+^ NK cells in the bone marrow are shown in **(F)** Six mice per group obtained from two independent experiments were used for **(B-F)**. Statistically significant differences between the groups were analyzed with an unpaired t test. All data are presented as mean values ± SEM.

In our previous analysis we found a correlation between activation and transferrin receptor expression as well as iron uptake of NK cells after IFNα stimulation (**Figures 1E, F, H**). Building on insights from our *in vitro* studies, the second part of our investigation examines the impact of dietary iron deficiency on NK cell functionality *in vivo*. Specifically, we aimed to determine how a low-iron diet affects the antiviral response of NK cells in mice infected with FV, revealing critical implications for immune performance under conditions of iron scarcity. We hypothesized that iron is essential for NK cell functions and the control of viral loads in virus infection. While viruses require iron for their replication and survival, NK cells also depend on iron for their activation and optimal function, creating a complex interplay where iron availability can simultaneously benefit both the pathogen and the host’s immune defense. To address the question whether diet-induced iron levels reduce or enhance viral replication, we fed mice for four weeks with low iron or control chow (**Figure 2A**). Next, we infected these mice with FV for 7 days. The analysis of serum iron showed a significant decrease in mice fed with low iron chow (low Fe) compared to mice fed with control (Ctr) diet (**Figure 2B**). FV mainly infects erythroid precursor cells (Ter119^+^), granulocytes (Gr1^+^), macrophages (F4/80^+^) as well as dendritic cells (CD11c^+^) (35). To elucidate the role of iron on FV target cells, we analyzed cell numbers of these cells in the bone marrow. We did not detect differences in the numbers of FV target cells in low iron fed animals compared to control diet fed mice (**Figure 2C**).

The bone marrow is the most active site of iron utilization in the body (17) and it has been shown that NK cells located in the bone marrow are very sensitive to alterations in iron availability (8). Thus, we analyzed FV replication in the bone marrow of infected animals, which were either fed with control diet (Ctr) or low iron containing diet (low Fe) (**Figure 2D**). Viral replication was significantly increased in animals fed with low iron diet compared to control diet-fed mice. Next, we analyzed the percentages of bone marrow NK cells in control or low iron diet fed animals (**Figure 2E**), which was comparable between both groups. By analyzing GzmB expression in NK cells from the bone marrow we found no differences in the expression of GzmB on NK cells, but a significantly decreased proportion of GzmB^+^ NK cells in animals fed with low iron diet (**Figure 2F**). This suggests that variations in viral loads are attributed to altered elimination of FV-infected cells, rather than solely to differences in the proliferation patterns of FV target cells. Collectively, iron deficiency increased viral replication and decreased GzmB in NK cells implicating a primary role of iron for immune activation rather than in FV replication.

### Improved NK cell metabolism and function after iron supplementation

**Figure 3:**
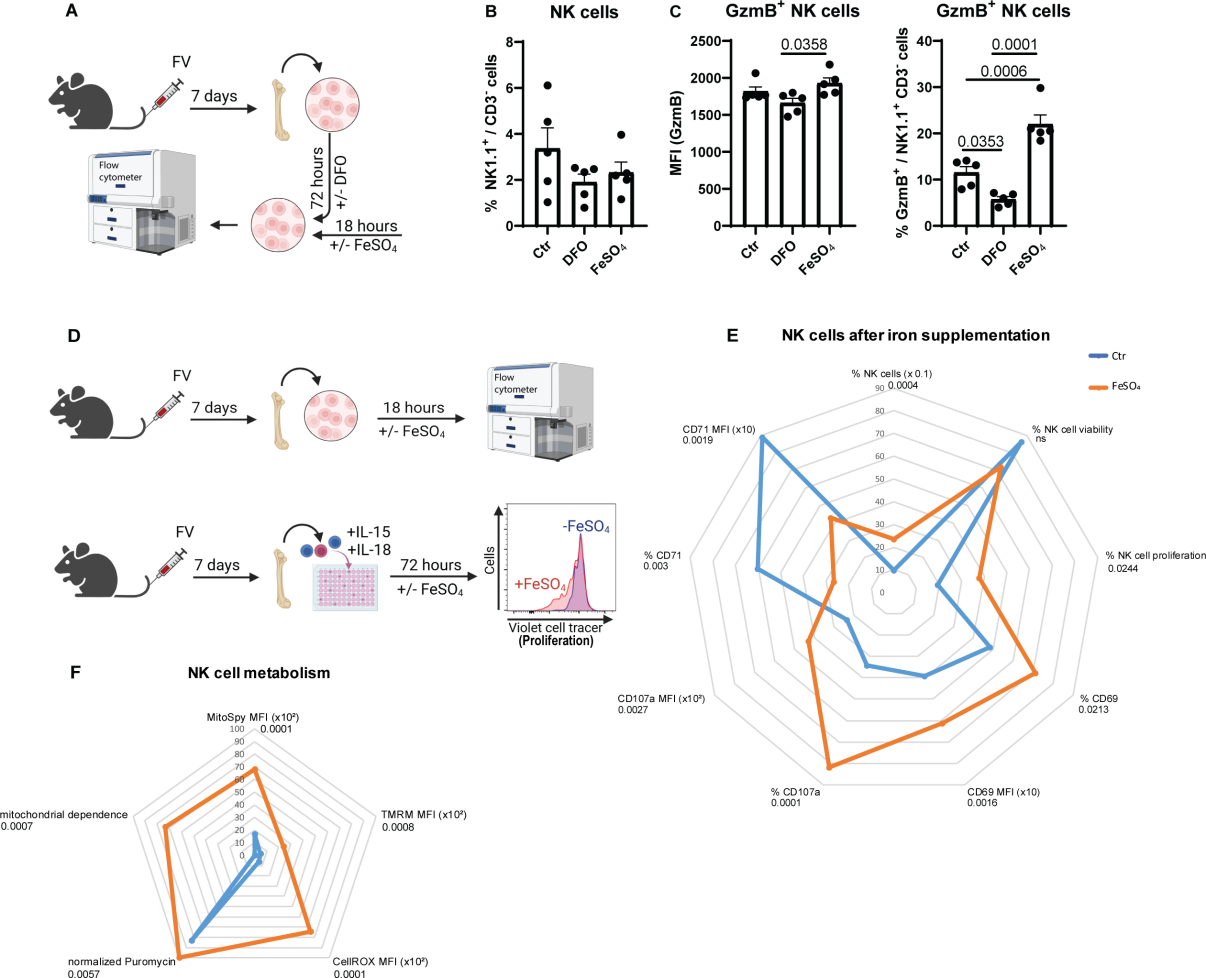
Augmented NK cell functions and mitochondrial polarisation after iron supplementation. C57BL/6 mice were infected with 40,000 SFFU of FV for 7 days and bone marrow cells were harvested. Cells were treated for 72 hours with DFO (100 µM) to reduce iron levels or left untreated. FeSO_4_ (200 µM) was added for the final 18 hours to separate wells **(A)**. NK cells were analyzed by flow cytometry **(B)**. In **(C)**, frequencies and GzmB expression of NK cell was determined. Five mice per group drawn from two independent experiments were analyzed by ordinary one-way ANOVA. Single cell suspensions were treated with or without FeSO_4_ for 18 hours (200 µM) or 72 hours (proliferation) **(D)** and analysed for NK cell numbers (Mann-Whitney test), NK cell viability (unpaired t test), proliferation (50 µM, unpaired t test), activation (CD69, % unpaired t test, MFI Mann-Whitney test) degranulation (CD107a, unpaired t test) and CD71 expression (unpaired t test) **(E)**. NK cell proliferation was analysed from six mice of three independent experiments, NK cell viability from fourteen mice of six independent experiments. In **(F)**, mitochondrial mass of NK cells (MitoSpy, unpaired t test), polarisation (TMRM, Mann-Whitney test), oxidative stress (CellROX, Mann-Whitney test), mitochondrial dependence (unpaired t test, 4 mice) as well as normalized puromycin levels (unpaired t test, 4 mice) as radar chart. Nine mice per group drawn from four independent experiments were used for analyses, if not indicated otherwise. ns = not significant.

As shown above, insufficient iron levels decreased NK cell’s GzmB expression and increased viral loads (**Figure 2**). To demonstrate that iron deficiency affects NK cells similarly *in vitro* as observed in our *in vivo* experiments with a low iron diet (**Figure 2**), we employed deferoxamine (DFO), an iron chelator, to induce iron deficiency in cell culture conditions and on the other hand used iron sulfate (FeSO_4_) to increase the iron *in vitro* (**Figure 3A**). Similar to the analyses of the low dietary iron treatment of mice (**Figures 2E, F**), we analyzed frequencies of NK cells (**Figure 3B**) and GzmB^+^ NK cells (**Figure 3C**), which were obtained from FV-infected animals (7 dpi) after *in vitro* stimulation with DFO or FeSO_4_. While we did not find differences in percentages of NK cells between untreated, DFO- or FeSO_4_-treated groups (**Figure 3B**), DFO significantly decreased the frequencies of GzmB^+^ NK cells, while FeSO_4_ treatment increased the percentage of GzmB^+^ NK cells (**Figure 3C**). The MFI of GzmB^+^ NK cells was not significantly altered by the DFO treatment, consistent with data from *in vivo* experiments (**Figure 3C**). These findings demonstrate that the low dietary iron experiments *in vivo* and the DFO/FeSO_4_ condition *in vitro* were well comparable. We further elucidated if iron supplementation can increase the NK cell activation as well as their metabolism, which could be important to improve the treatment of viral infections and cancers. Therefore, NK cells from FV-infected mice (7 dpi) were stimulated with FeSO_4_ and analyzed for their effector phenotype (18 hours; **Figure 3D**). NK cell proliferation was assessed by tracking cell divisions through the progressive dilution of a fluorescent dye (72 hours; **Figure 3D**). NK cell numbers were significantly increased after iron treatment, without any observable differences in NK cell viability (**Figure 3E**). Instead, an increased frequency of proliferation was detectable in iron-treated NK cells. Increased expression and frequencies of the activation marker CD69 and degranulation marker CD107a in iron-supplemented NK cells were identified (**Figure 3E**) and shown as representative histogram in **Supplementary Figure 1** or as bar graphs in **Supplementary Figure 2**. Interestingly, iron supplementation decreased the expression and frequency of CD71 in NK cells (**Figure 3E**). Upon infection, NK cells rearrange their mitochondria to fuel their increased demands for energy (8) and iron is involved in multiple mitochondrial processes (18). Hence after iron stimulation, NK cells enhanced the expression of MitoSpy, an indicator for mitochondrial mass (**Figure 3F**). Additionally, we detected an increased membrane polarization measured by accumulation of TMRM dye into mitochondrial membrane (**Figure 3F**). Interestingly, iron supplementation also increased the cellular stress in NK cells, as demonstrated by increased CellROX levels (**Figure 3F**). Notably, our investigation revealed that NK cell responses following murine cytomegalovirus (MCMV) or herpes simplex virus type 1 (HSV-1) infection exhibited consistent alterations in response to iron supplementation (**Supplementary Figure 3**, **Supplementary Figure 4**). We then investigated whether extracellular iron could enhance NK cell function by boosting their metabolic activity (7 dpi, FV). For this, we employed SCENITH, a single-cell technique that enables immunometabolic characterization through the analysis of cellular protein translation (36). Our findings demonstrate that NK cells exhibited increased protein translation rates (**Figure 3F**), as evidenced by puromycin incorporation, following iron treatment. Additionally, upon iron stimulation, NK cells showed an increased dependence on oxidative phosphorylation, also measured by SCENITH (**Figure 3F**). These data collectively show that NK cell functions and their mitochondrial machinery can be improved by iron supplementation.

### Augmented NK cell cytotoxicity after iron supplementation

**Figure 4:**
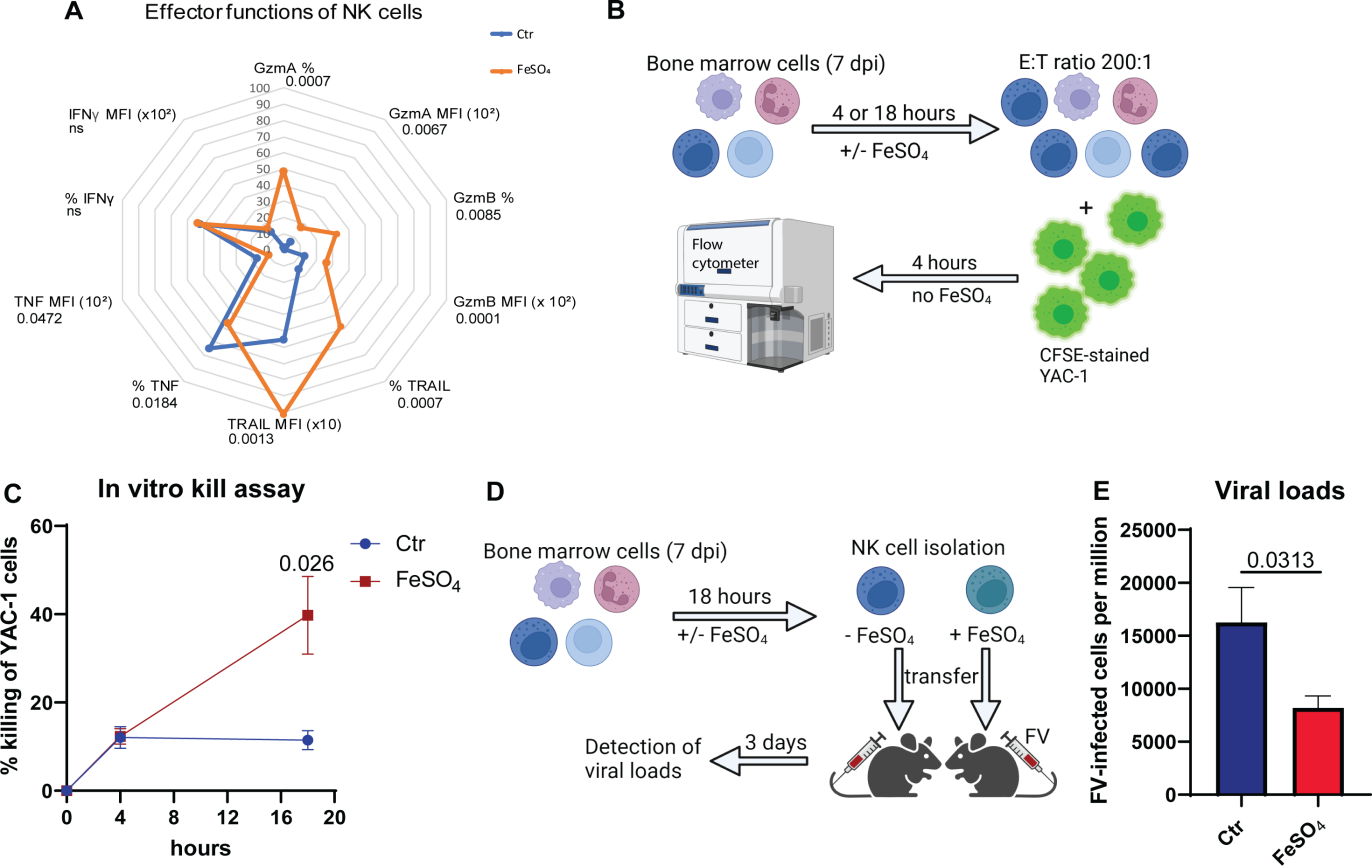
Iron supplementation increased the killing of NK cells. C57BL/6 mice were infected with 40,000 SFFU of FV for 7 days and bone marrow cells were harvested. Single cell suspensions were treated with or without FeSO_4_ for 18 hours (200 µM). Single cell suspensions were analyzed for cytotoxicity-related molecules such as GzmA (unpaired t test), GzmB (unpaired t test), TRAIL (unpaired t test) and cytokines such as IFNγ and TNF (unpaired t test, n_Ctr_ = 9, n_FeSO4_ = 8) **(A)**. Nine mice per group drawn from four independent experiments were used for analyses, if not indicated otherwise. Single cell suspensions were treated with or without FeSO_4_ (200 µM) for 4 or 18 hours **(B)**. Cells were washed and coincubated with CFSE-stained YAC-1 cells in an effector: target cell (E:T) ratio of 200:1. 4 hours later, killing of YAC-1 cells was analyzed at flow cytometry (BD Canto II) **(C)**. Ten mice per group from four independent experiments (4 hours) and six mice from three independent experiments (18 hours) were used and analyzed by Mann-Whitney test. Bone marrow cells were treated with or without FeSO_4_ and NK cells were isolated thereof **(D)**. 0.5 x10^6^ NK cells were transferred i.v. into mice, infected with FV at the same day. Viral loads were determined in the bone marrow **(E)**. Six (untreated) or seven (FeSO_4_) mice per group obtained from two independent experiments were used and analyzed by an unpaired t test. All data are presented as mean values ± SEM. E:T = effector: target; ns = not significant.

Because iron supplementation can potentially enhance NK cell responses, particularly in the context of iron deficiency or increased iron demand during infections, parameters determining the NK cells cytotoxicity were analyzed in **Figure 4A**. We examined the cytotoxicity-associated markers GzmA, GzmB and TRAIL in iron-treated NK cells compared to untreated cells (**Figure 4A**). Percentages and median fluorescence intensities (MFI) of GzmA^+^, GzmB^+^, as well as TRAIL^+^ NK cells were significantly increased after iron stimulation. Iron stimulation of NK cells from MCMV or HSV-1 showed similar results (**Supplementary Figure 3**, **Supplementary Figure 4**). Surprisingly, cytokines expressed by NK cells, such as IFNγ or TNF, were rather decreased (TNF) or unchanged (IFNγ) upon iron supplementation (FV, **Figure 4A**). Next, we examined the cytotoxicity of NK cells in an *in vitro* kill assay. NK cell killing was analyzed after 4 or 18 hours of FeSO_4_ treatment. To measure the killing capacity of iron-pretreated NK cells, bone marrow cells were cocultured with CFSE-stained NK target cells (YAC-1) for 4 hours. After the coincubation, target cells were quantified by flow cytometry (**Figure 4B**) and NK cell-mediated killing was calculated (**Figure 4C**). The killing capacity of NK cells did not differ between the untreated and treated group after 4 hours of FeSO_4_ stimulation. However, after 18 hours of FeSO_4_ treatment, killing was 3.5 times higher in the iron-stimulated group compared to non-supplemented group. Next, we analyzed the NK cell killing *in vivo* after FeSO_4_ stimulation of NK cells *in vitro*. Therefore, we extracted bone marrow cells of FV-infected mice (7 dpi) and stimulated them with FeSO_4_ for 18 hours. Untreated bone marrow cells (-FeSO_4_) served as control. NK cells were then isolated from treated and untreated bone marrow cells using magnetic beads and 0.5 x 10^6^ NK cells were transferred into naïve mice, which were subsequently infected with FV (**Figure 4D**). Viral loads were determined in the bone marrow at 3 dpi (**Figure 4E**). Mice that received iron-treated NK cells exhibited significantly reduced viral loads compared to mice that received untreated NK cells. These data demonstrate a beneficial and stimulatory effect of iron on the NK cell cytotoxicity *in vitro* and *in vivo* resulting in improved FV control.

## Discussion

Iron is crucial for numerous biological processes, including DNA synthesis, energy production and oxygen transport, with approximately 70% of the body’s iron is incorporated into red blood cell hemoglobin (17). Thus, across ages a common consequence of iron deficiency is anemia, which is defined by hemoglobin (Hb) levels <12.0 g/dL in women and <13.0 g/dL in men according to World Health Organization (37). On the other hand, excessive iron accumulation can result in heightened oxidative stress and DNA damage, contributing to genetic instability and the development of cancer (38). Thus, it is crucial to maintain strict regulation of iron levels. In immune cells, iron is essential for their function and proliferation, as it is involved in processes such as gene regulation and metabolism, highlighting its importance in maintaining a robust immune response (20). However, iron is also crucial for viral replication, transcription and assembly, making it a key nutrient that viruses compete for within host cells (16). Therefore, it was extremely uncertain whether iron deficiency or iron supplementation would influence viral replication or NK cell functionality. In this study, we demonstrate that inadequate iron supplementation in diet resulted in increased viral replication, in this case of Friend retrovirus (FV), and decreased frequencies of GzmB^+^ NK cells. Iron supplementation did not solely elicit the NK cell proliferation and activation, but also degranulation and cytotoxicity.

Most viral infections rapidly induce type I IFN responses leading to the transcription of IFN-stimulated genes (5). There are several IFNα subtypes, which have different antiviral activities. For IFNα11, it has been shown before that NK cells are extremely sensitive to its stimulation and showed increased killing (6). Our data demonstrate that IFNα11 enhances the expression of the transferrin receptor CD71, that is important for the uptake of transferrin-bound iron, which may contribute to the antiviral activity of IFNs by modulating iron metabolism, a critical factor for viral replication and immune responses.

FV and HIV are both retroviruses, with FV affecting mice and HIV infecting humans. Unlike HIV, which replicates primarily in CD4^+^ T cells, FV replicates predominantly in erythrocyte precursors, granulocytes, macrophages and DC as well as dividing cells, leading to distinct pathological outcomes (35, 39). Despite these differences in disease progression, both viruses share similarities in their basic retroviral structure and replication cycle, including the use of reverse transcriptase to convert their RNA genome into DNA for integration into the host cell’s genome. FV has been extensively studied as a model for retroviral infections and immune responses, providing valuable insights into host-pathogen interactions that can be applied to HIV research (26, 29). Interestingly, similar cell numbers of FV target cells were detectable after low iron diet suggesting no direct effect of low iron diet on cell expansion. The human body comprises about 4 grams of iron, and despite only absorbing about 1 milligram daily, it proficiently recycles iron to maintain required levels (17). Despite rapid changes and turnover in iron utilization, plasma iron levels are largely constant (17). Notably, the administration of a low-iron diet resulted in a modest yet statistically significant decrease in serum iron concentrations. Viruses such as the complex retrovirus HIV modulate cellular and systemic iron homeostasis to benefit from iron resulting in reduced hemoglobin concentrations in hosts (40). Drakesmith et al. and Madrid et al. examined the role of the HIV accessory protein Nef and the expression of the transferrin receptor CD71 (41, 42). They reported that Nef impaired the recycling of CD71 to the cell surface, which correlated with reduced iron uptake and iron deficiency. IFNα stimulation and acute infection with FV, a simple murine retrovirus, resulted in an upregulation of CD71 (8), and only iron supplementation reduced the CD71 expression on NK cells similar to CD71 on CD4^+^ T cells (concentration of iron supplementation not mentioned) suggesting a degree of iron saturation and compensatory mechanism to restrict iron uptake (43). In people living with HIV (PLWH) iron deficiency is associated with increased mortality and unsuppressed viral loads (44). In our murine model for retroviral infection, low dietary intake of iron was associated with increased viral loads in FV infection and, interestingly, decreased cytotoxicity of NK cells, as shown by GzmB expression. Levels of NK cells producing cytokines were not altered or even lower after iron supplementation, which is in line with a diminished differentiation of CD4^+^ T helper type 1 (Th1) cells and the expression of IFN-γ after an iron loading before bacterial infection (45). These findings indicate that iron plays a more critical role in the cytotoxic function of immune cells, like NK cells, than in viral replication. Others have shown that HIV infection lead to increased hepcidin levels, thus resulting in reduced serum iron concentrations (46). Increased hepcidin titers are associated with augmented mortality in PLWH, linking iron metabolism, anemia and HIV (47).

Iron accumulation and overdose consequently result in heightened oxidative stress and DNA damage, contributing to genetic instability and carcinogenesis (38). High levels of cellular iron generate reactive oxygen species (ROS), particularly hydroxyl radicals, which destabilize and degrade the phospholipid membrane. This process triggers ferroptosis, an iron-dependent mechanism of cell death (48). Interestingly, we show that iron therapy resulted in increased mitochondrial fitness and mitochondrial dependence, but also increased the cellular stress in NK cells after acute infection with the murine FV. Increased production of reactive oxygen species and stress in high iron conditions was also shown for various cells, e.g., hepatic HepG2 cells, chondrocytes, neuronal cells (49–51). It has been shown that iron overload is associated with enhanced progression of disease and poor prognosis, e.g. in HIV, hepatitis B virus or hepatitis C infections (16). Interestingly, accumulation of iron in macrophages was shown to enhance HIV replication and was associated with a poor outcome in PLWH (52). In our murine study, we fed NK cells *in vitro* with additional iron and analyzed their fitness and cytotoxic function. Iron-treated NK cells were highly activated, degranulated and eliminated target cells proficiently. In 2024, Abioye et al. showed a different outcome of iron supplementation (60 mg elemental iron in 200 mg ferrous sulfate daily) depending on therapy-naïve individuals or people on antiretroviral therapy (ART) (44). Iron-supplemented people who continue ART had an increased risk of unsuppressed viral load. Nevertheless, in therapy naïve individuals, iron supplementation was not associated with mortality whereas there was a lower incident of mortality among people on ART (44). Hence, iron supplementation in iron-deficient individuals can have complex effects on immune function and infection outcomes. While repletion of iron stores can restore the function of iron-dependent enzymes and improve overall health, it can also reactivate latent infections and promote the growth of pathogens. This dual role of iron highlights the need for careful management of iron levels in patients, particularly those with chronic infections or immune deficiencies. Thus, it is tempting to speculate that fueling the iron metabolism of NK cells might promote NK cells activity and viral elimination resulting in better clinical outcomes.

## Data Availability

The raw data supporting the conclusions of this article will be made available by the authors, without undue reservation.
