# Supplementary material for: Iron improves the antiviral activity of NK cells
Source: Front Immunol. 2025 Jan 14;15:1526197. doi: 10.3389/fimmu.2024.1526197 (PMC11772171; doi:10.3389/fimmu.2024.1526197)
Supplement: Supplementary file 1 [file DataSheet1.docx]

Supplementary Material

**
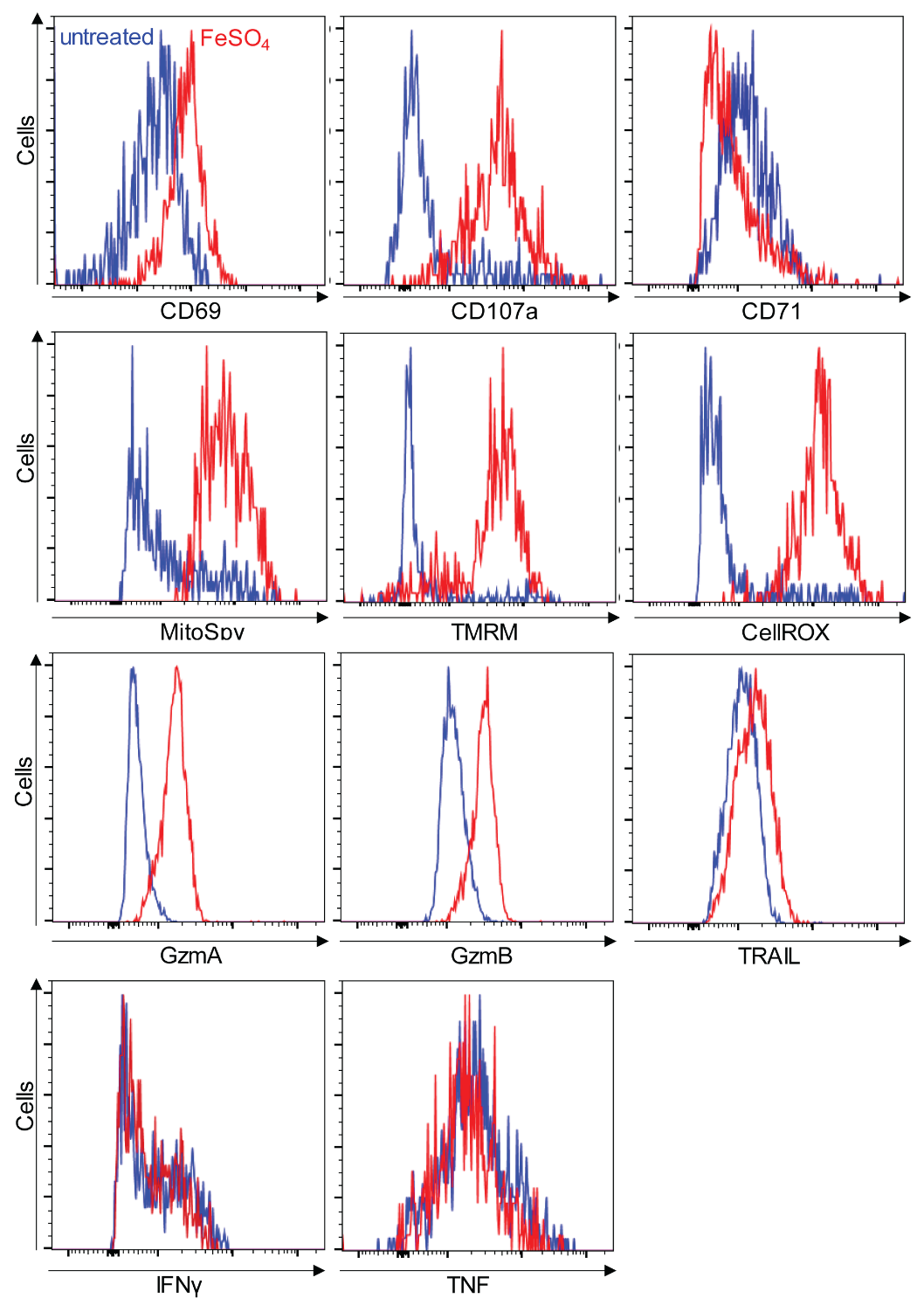
**

**Supplementary Figure 1.** **Representative histograms of untreated and iron-treated NK cells.** C57BL/6 mice were infected with 40,000 SFFU of FV for 7 days and bone marrow cells were harvested. Single cell suspensions were treated with (shown in red) or without (shown in blue) FeSO_4_ (200 µM) for 18 hours. Flow cytometry stainings were performed and analysed at BD Canto II.


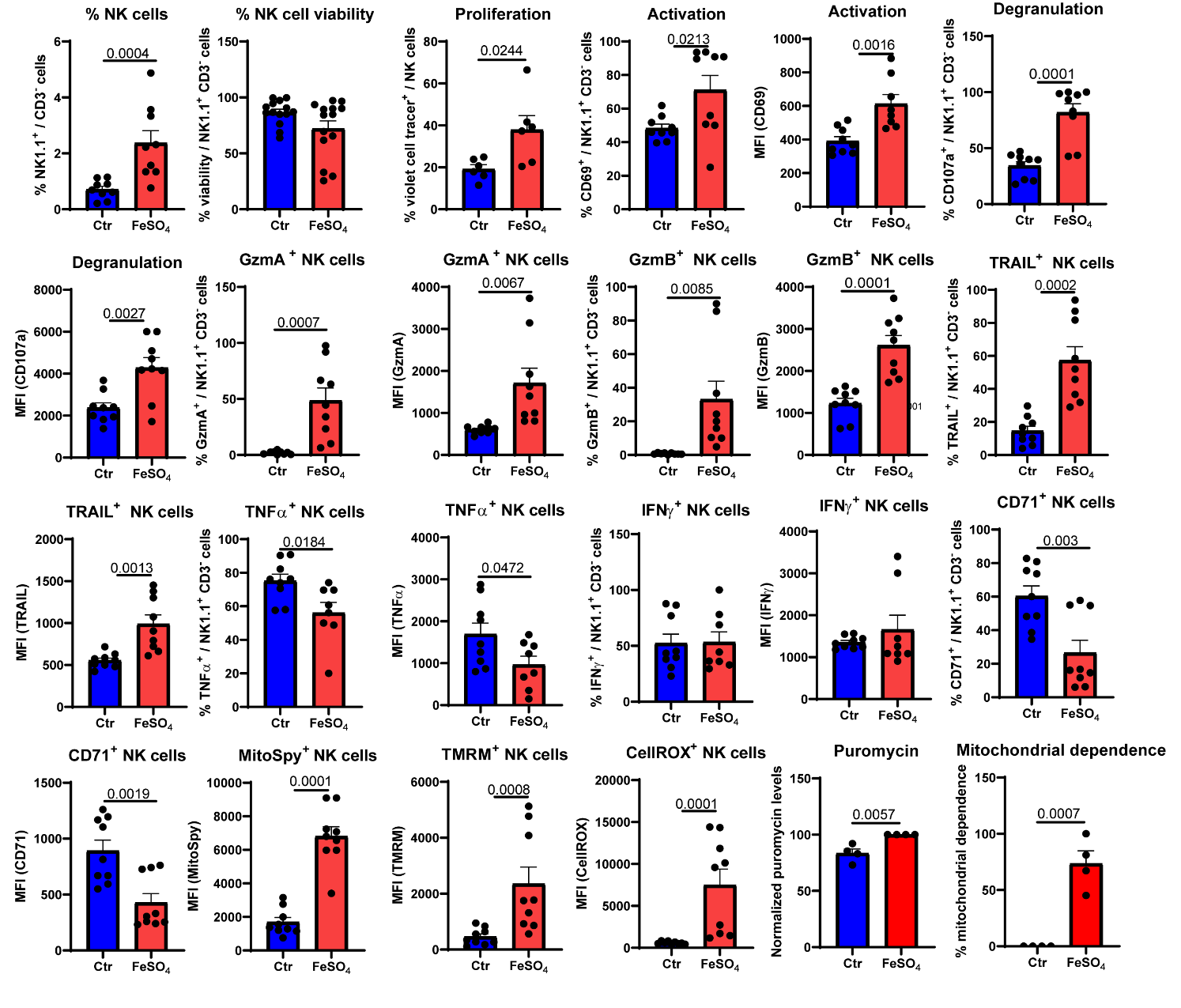


**Supplementary Figure 2.** **Bar graphs of untreated and iron-treated NK cells.** C57BL/6 mice were infected with 40,000 SFFU of FV for 7 days and bone marrow cells were harvested. Single cell suspensions were treated with (red) or without FeSO_4_ (blue) for 18 hours (200 µM) or 72 hours (50 µM, proliferation) and analyzed for NK cells (Mann-Whitney test), NK cell viability (unpaired t test), proliferation (unpaired t test), activation (CD69, % unpaired t test, MFI Mann-Whitney test) and degranulation (CD107a, unpaired t test). NK cell proliferation was analysed from six mice of three independent experiments, NK cell viability from fourteen mice of six independent experiments. CD71 expression (unpaired t test) on NK cells is displayed as well as the analysis of mitochondrial mass (MitoSpy, unpaired t test), polarisation (TMRM, Mann-Whitney test), oxidative stress (CellROX, Mann-Whitney test), mitochondrial dependence (unpaired t test, 4 mice) as well as normalized puromycin levels (unpaired t test, 4 mice). Single cell suspensions were analyzed for cytotoxicity-related molecules such as GzmA (unpaired t test), GzmB (unpaired t test), TRAIL (unpaired t test) and cytokines such as IFNγ and TNFα (unpaired t test, n_Ctr_ = 9, n_FeSO4_ = 8). Nine mice per group drawn from four independent experiments were used for analyses, if not indicated otherwise.


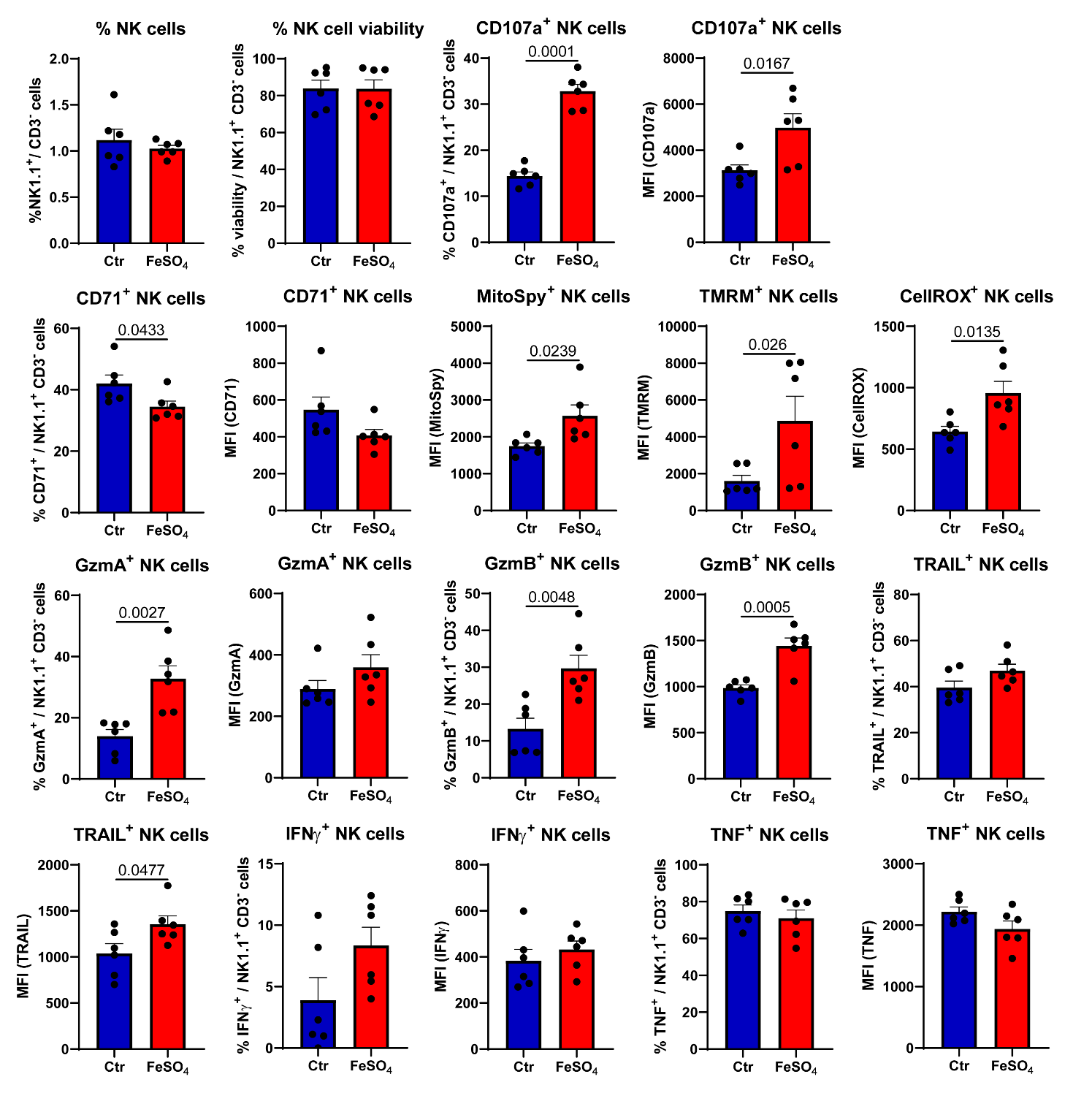


**Supplementary Figure 3.** **Increased functionality and enhanced metabolism in iron-treated NK cells after MCMV infection.** C57BL/6 mice were infected with 2x10^5^ PFU for 8 weeks and bone marrow cells were harvested. Single cell suspensions were treated with (red) or without FeSO_4_ (blue) for 18 hours (200 µM) and analyzed for NK cells, NK cell viability, degranulation (CD107a), CD71 expression, mitochondrial mass (MitoSpy), polarisation (TMRM) and oxidative stress (CellROX), cytotoxicity-related molecules such as GzmA, GzmB, TRAIL and cytokines such as IFNγ and TNFα Six mice per group drawn from two independent experiments were used for analyses. Statistically significant differences between the two groups were analyzed by an unpaired t test or Mann-Whitney test (TMRM).


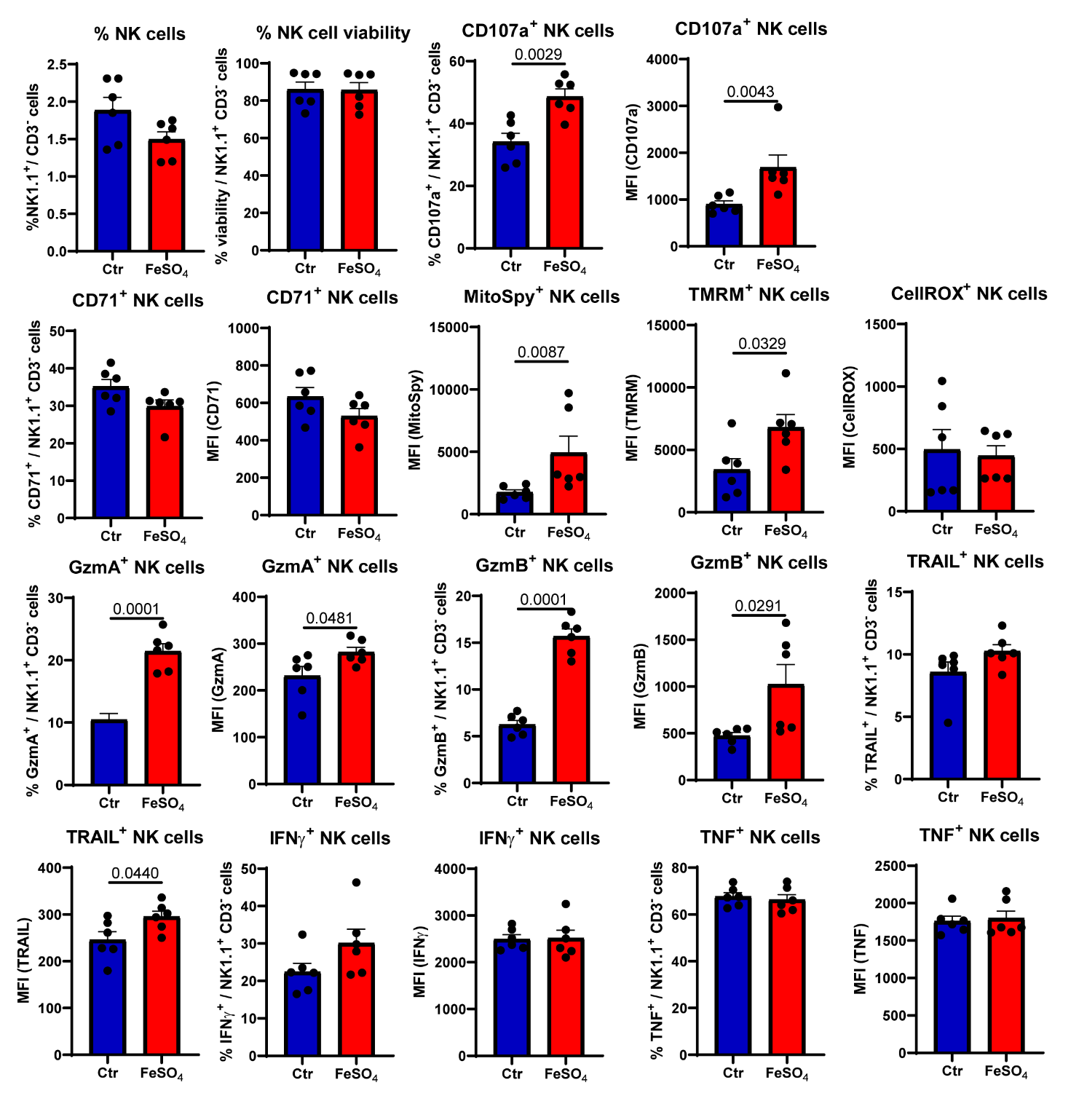


**Supplementary Figure 4.** **Increased functionality and enhanced metabolism in iron-treated NK cells after HSV-1 infection.** C57BL/6 mice were infected with 5x10^6^ TCID50 HSV-1 for 8 weeks and bone marrow cells were harvested. Single cell suspensions were treated with (red) or without FeSO_4_ (blue) for 18 hours (200 µM) and analyzed for NK cells, NK cell viability, degranulation (CD107a), CD71 expression, mitochondrial mass (MitoSpy), polarisation (TMRM) and oxidative stress (CellROX), cytotoxicity-related molecules such as GzmA, GzmB, TRAIL and cytokines such as IFNγ and TNFα Six mice per group drawn from two independent experiments were used for analyses. Statistically significant differences between the two groups were analyzed by an unpaired t test or Mann-Whitney test (MitoSpy, MFI CD107a).
